# Psychometric Validation of the Connectedness to the LGBT Community Scale among Black Sexual Minority Men Living with HIV

**DOI:** 10.1007/s10900-024-01392-z

**Published:** 2024-08-27

**Authors:** Rodman Turpin, Derek T. Dangerfield, Temitope Oke, Roland J. Thorpe, DeMarc A. Hickson

**Affiliations:** 1 Department of Global and Community Health, College of Public Health, George Mason University, Fairfax, VA, USA; 2 Us Helping Us, People Into Living, Inc., Washington, DC, USA; 3 Department of Prevention and Community Health, Milken School of Public Health, George Washington University, Washington, DC, USA; 4 Program for Research on Men’s Health, Hopkins Center for Health Disparities Solutions, Johns Hopkins Bloomberg School of Public Health, Baltimore, MD, USA

**Keywords:** Methods, Community, Social, Race, Sexuality, HIV

## Abstract

**Purpose:**

LGBTQ + community connectedness is generally a protective health factor for sexual and gender minorities. However, existing scales have not been validated among Black sexual minority men living with HIV (SMMLWH), who face unique marginalized experiences that disproportionately impact several health outcomes compared to the general LGBT + community. We validated the Connectedness to the LGBT Community Scale among Black SMMLWH.

**Methods:**

We validated the 9-item Connectedness to the LGBT Community Scale from Frost and Meyer using preliminary data from a cohort of Mid-Atlantic Black SMMLWH (*n* = 650). Factor analysis and intercorrelations were conducted to assess unidimensionality, and Cronbach’s alpha was measured for reliability. Correlations and cumulative ordinal regression models were generated using internalized homophobia, hopelessness, depression, HIV stigma, social support, and resilience as criterion constructs. Models were adjusted for sociodemographic and behavioral characteristics.

**Results:**

The Connectedness to the LGBT Community Scale demonstrated high internal consistency (alpha = 0.948) and strong item intercorrelation with a single factor structure. The scale was associated with all criterion measures before and after adjustment, including lower internalized homophobia (aCOR = 0.19, 95% CI 0.15–0.25), lower hopelessness (aCOR = 0.53, 95% CI 0.41–0.68), lower HIV stigma (aCOR = 0.58, 95% CI 0.47–0.72), and lower depression (aCOR = 0.61, 95% CI 0.50–0.75). The scale was also associated with greater social support (aCOR = 2.38, 95% CI 1.91–2.97) and resilience (aCOR = 2.53, 95% CI 2.03–3.15).

**Conclusion:**

The Connectedness to the LGBT Community Scale is a valid measure for use among Black SMMLWH. Future studies should explore relationships between community connectedness and HIV care outcomes and quality of life among Black SMMLWH.

## Introduction

Despite overall advancements in HIV treatment and prevention, Black sexual minority men living with HIV (SMMLWH) continue to face greater challenges in care retention, treatment adherence, and viral suppression than others, primarily due to the culmination of stigma, discrimination, and less access to health services, among other socioecological factors [1]. Connection to the LGBTQ + community is a crucial support system that provides emotional support, resources, and advocacy, all of which are needed to improve HIV care outcomes and quality of life for Black SMMLWH [2–5]. For Black SMMLWH, connection to the LGBT + community could be an important buffer against negative sexual, behavioral, and physical health outcomes, including depression and suicidality [6–8]. However, measures of LGBT + community connectedness are not well validated in this population.

The theoretical basis for LGBT + community connectedness as a protective factor primarily revolves around several key social, ecological, and psychological theories that explain how connections across LGBT + community networks have profound improvement on health outcomes and quality of life. For example, Social Capital Theory suggests that social networks provide access to financial, emotional, and institutional resources that are crucial for individuals’ social, economic, and physical well-being [9, 10]. Additionally, Minority Stress Theory suggests that minority groups such as LGBT + individuals experience a higher level of stress related to their marginalized identitieswhich can lead to adverse health outcomes [11]. Therefore, for sexual minority individuals, LGBT + Community connectedness can serve as a vital resource for navigating societal challenges, accessing information about health and human rights, and emotional support, particularly in contexts of identity, marginalization, and accessing resources [4, 9]. The presence of enduring relationships and mutual trust within a community fosters cohesion and leads to improved health results over time. One study showed that social support could moderate the effects of school bullying on mental health through mechanisms like reducing internalized homophobia among lesbian, gay, and bisexual college students [12]. Collectively, the literature underscores the importance of community connectedness in mitigating mental, sexual, and physical health disparities among LGBTQ + populations. This may be especially salient for Black SMMLWH [7, 10, 13–15].

Although measures of LGBT + community connection are limited, one that captures this concept is the Connectedness to the LGBT Community Scale [4]. This measure has not been studied in Black SMMLWH, which limits inferences regarding the role of this construct among this highly vulnerable population. This warrants exploration of the validity of this measure among this population for multiple reasons. First, Black SMMLWH experience a unique social context than other LGBT + or HIV-affected populations given their multiple identities (e.g., Black and SMMLWH) [3, 7, 15]. Second, the Black LGBT community is an important channel for health promotion messaging and access, including HIV prevention, treatment and care services [5, 16–18]. Disconnection from the LGBT community may present important multi-level barriers for Black SMMLWH; this has considerable implications given the disproportionate burden of HIV among Black SMM [19, 20]. Moreover, measures of LGBT community connectedness can be useful for research efforts, both in needs assessment, the development of interventions, and implementation science [3, 6, 16, 21]. These measures can improve our understand of how community improves the health and well-being of Black SMMLWH [6, 9, 14, 22, 23]. Having a valid tool for measuring LGBT community connectedness is essential for achieving health equity in this population.

Therefore, to fill these gaps in the literature, the purpose of this study is to validate the Connectedness to the LGBT Community Scale among Black SMMLWH. We hypothesized sufficient internal consistency in our measure, positive relationships between the Connectedness to the LGBT Community Scale and both social support, and resilience, and negative relationships between the Connectedness to the LGBT Community Scale and internalized homophobia, hopelessness, depression, and HIV stigma.

## Methods

### Sample and Recruitment

Analyses utilized preliminary data from a digital/virtual cohort feasibility study of Mid-Atlantic BSMMLWH. Recruitment involved a combination of active and passive strategies [24–27]. For example, active recruitment included reaching out to individuals from previous studies and clients from both community-based venues and our community partner organizations who provided consent to be contacted for future studies. Passive recruitment involved placing ads on social media and geosocial network platforms including X (formerly known as Twitter) Jack’d. Individuals also refereed others within their social network to the study. Participants were eligible based upon the following self-reported criteria: Black/African American race; male sex at birth; man gender, same-sex attraction to men; living with HIV; residing in Maryland, Pennsylvania, or Washington, D.C. Participants provided electronic informed consent completed an online survey assessing a range of demographic, personal, and contextual factors. Following the survey, participants were provided a $75 electronic Amazon gift card. All activities were approved by the George Washington University Institutional Review Board (IRB # 10121).

### Measures

#### Connectedness to the LGBT Community

The Connectedness to the LGBT Community Scale [4] is an 8-item measure that was originally developed and validated among LGBT people living in New York in 2011. Items capture several aspects of community connection, including emotional social cohesion (e.g., “You feel a bond with NYC’s LGBT community”) and a sense of community empowerment (e.g., “If we work together, gay, bisexual and lesbian people can solve problems in NYC’s LGBT community”). As we are examining these questions in a context outside NYC, we modified items to omit that specification (e.g., “You feel a bond with NYC’s LGBT community” was changed to “You feel a bond with the LGBT community”). Since we are studying this among SMM, we also added an item: “You feel a bond with other gay men”. For this study, we used a 9-item scale for all psychometric analyses.

#### Criterion Measures and Covariates

Criterion measures of interest included internalized homophobia, measured using Herek and Glunt’s 9-item Internalized Homophobia scale [28], hopelessness measured using the 5-item Hopelessness Scale [29], depression measured using the CESD-20 [30], HIV stigma measured using the 10-item HIV stigma scale [31], social support measured using the 3-item Social Support Questionnaire [32], and resilience measured using the 6-item Brief Resilience Scale [33]. Covariates included age, state, education, income, housing status, gender of sexual partners, sexual position, and relationship status.

### Statistical Approach

Analyses were conducted in two steps. First, analysis involved extracting factors and testing dimensionality using the Connectedness to the LGBT Community Scale items. Associations with covariates were also tested. We then assessed the summative scale reliability, including convergent validity. Second, criterion validity was assessed through bivariate and regression analyses between the Connectedness to the LGBT Community Scale and all criterion measures.

### Missing Data

Missingness across all variables was low, with less than 5% missingness for all items. Intrascale stochastic imputation was used to impute missing values, using non-missing items within scale to impute missing items. Imputation was used because it allows for consistency of our analytic sample and does not have a substantial impact on our ability to identify subscales, since there are relatively few imputed values. Our scales all demonstrated acceptable, good, or excellent internal consistency (Cronbach’s alpha > 0.70), supporting the validity of this means of imputation.

### Bivariate Analyses

Bivariate analyses included utilizing Spearman’s Rank-Sum correlations for ordinal covariates (i.e., age, education, income), Kruskal-Wallis tests for multicategorical covariates (i.e., state, sexual position), and Cochran-Armitage tests of trend for binary covariates (i.e., housing status, gender of sexual partners, relationship status).

### Extraction of Factors and Testing Dimensionality

First the factor structure of the Connectedness to the LGBT Community Scale was examined using exploratory factor analysis (EFA) with maximum likelihood factoring. Next, confirmatory factor analysis comparing models with the previously identified number of factors from the EFA to those with a fixed different number of factors. The goodness-of-fit index, root mean square error of approximation, and variance proportion explained to compare model fit. The model with the most ideal balance of goodness of fit and factor parsimony was selected; the presence of more than one factor suggests subscales.

### Summative Scale Reliability

Item correlation with the scale total score was used to determine how well each item correlates with the overall scale. The Cronbach alpha of the scale with each item removed was examined to determine how each item affects the internal consistency of the scale items. Items that reduced the overall internal consistency of the scale to less acceptable levels will be deleted from the summative scale. Cronbach alphas were considered acceptable at 0.7, good at 0.8, and exceptional at 0.9.

### Convergent Validity

To assess convergent validity of items, we tested for positive Spearman’s rank-sum correlations between all items used in our total scale. Identified subscales were tested for intercorrelations among each generated subscale using Spearman’s rank-sum correlations as well. Lack of correlation could indicate strong separate construct subdomains or that one or more subscales are not effectively measuring LGBT community connectedness [34].

### Criterion Validity

To assess criterion validity, the scale scores were compared to six criterion constructs: Internalized homophobia, hopelessness, depression, HIV stigma, social support, and resilience, as these have had well documented relationships with community connectedness in other populations [9, 12, 23]. First, we assessed Spearman’s rank-sum correlations between all measures. Next, we used cumulative ordinal regression models to test associations between a quartile increase in the Connectedness to the LGBT Community scale, and each of the 6 criterion outcomes. We generated both unadjusted cumulative odds ratios, and ratios adjusted for age, state, education, income, housing status, gender of sexual partners, sexual position, and relationship status.

### Quality Assurance

For regression analyses, leverages were used to assess outliers; no observations demonstrated unusually high leverages. Multicollinearity was tested by measuring the variance inflation factor (VIF) in each model. After removal of sexual position as a covariate, there was no evidence of collinearity in any of the models (All VIF < 5). We conducted all analyses in SAS 9.4 [35].

## Results

### Sample Characteristics

The distribution of social and demographic characteristics is shown in Table 1. Most participants were 25–34 (46.8%) or 35–44 years old (45.9%), and just under half report living in Washington, D.C. (46.8%), a third in Pennsylvania (46.8%), and 16.3% in Maryland. Most (85.7%) also had a 4-year college degree or more and 22.2% reported having a graduate degree. The median reported income was $60,000-$80,000 per year, and 85.7% reported having stable housing. Two-thirds self-identified as gay or same-gender loving (65.0%), and less than a third who have sex with men identified as straight or heterosexual (31.1%). Using the total scale score (mean 27, range 0 to 36), greater connectedness to the LGBT community was associated with older age, higher education, having only make sexual partners, gay / same-gender loving identity, top sexual positioning, and being partnered. Participants in Pennsylvania reported the highest connectedness to the LGBT community, while those in Washington, D.C. reported the lowest.

### Factor Analyses, Reliability, and Bivariate Associations

Descriptive statistics and factor loading for each item is shown in Table 2. Overall, participants generally endorsed LGBT community connectedness items; every item had a mean and median value of 3 (3=”Agree”). Exploratory factor analysis identified a single factor that explained most of the variance of all items (> 80%). Additionally, the 9-item scale demonstrated exceptionally high internal consistency (Cronbach’s alpha = 0.95). Each item was also tested for possible reduction by removing any items that would increase the overall internal consistency of the measure. Every single item contributed positively to the internal consistency of the measure. Thus, all items were retained in the final single factor item structure and there was no evidence to support creation of subscales. Confirmatory factor analysis supported this structure as well, with a GFI of 1, a RMSEA < 0.01, and the single primary factor explaining more than 80% of all variance across models with 1 to 5 fixed factors.

### Criterion Validity

Relationships between connectedness to the LGBT community and criterion constructs are displayed in Table 3. Connectedness to the LGBT community was very strongly associated with lower internalized homophobia, lower depression, lower HIV stigma, higher social support, and higher resilience (rho ranging from − 0.58 to −0.77, *p* < 0.01). It was also weakly, yet statistically associated with lower hopelessness (rho=−0.12, *p* < 0.05). Regression analyses (both unadjusted and adjusted) were consistent with these patterns (Table 4). After adjustment for age, state, education, income, housing status, gender of sexual partners, sexual position, and relationship status, all estimate patterns remained consistent. A quartile increase in LGBT community was associated with odds of internalized homophobia five times as low (aCOR = 0.19, 95% CI 0.15–0.25), hopelessness twice as low (aCOR = 0.53, 95% CI 0.41–0.68), HIV stigma twice as low (aCOR = 0.58, 95% CI 0.47–0.72), depression just over a third lower (aCOR = 0.61, 95% CI 0.50–0.75), social support over twice as high (aCOR = 2.38, 95% CI 1.91–2.97), and resilience over twice as high (aCOR = 2.53, 95% CI 2.03–3.15).

## Discussion

The Connectedness to the LGBT Community Scale was sufficiently internally consistent with a single factor structure and associated with all criterion measures in hypothesized directions. Specifically, greater LGBT community connection was associated with greater social support and resilience, and lower internalized homophobia, hopelessness, depression, and HIV stigma. Findings confirm many of the relationships between social connection and these factors in other populations [2, 3, 6, 7, 9, 21]. For example, one study found that feelings of social connectedness significantly mediated mental health outcomes among LGBT + individuals [2]. The relationships with all criterion constructs were not only remarkably strong, but maintained even after adjustment for a robust set of sociodemographic and relationship-related confounders. These strong relationships highlight the substantial importance of LGBT community connection to these health-related outcomes among Black SMMLWH. Given these strong criterion relationships, the high internal consistency, and the positive contributions of each item to the overall validity of the scale, our findings support the use of this measure in the complete, 9-item form.

Participants who were partnered, gay/same-gender loving, and reported having only male sexual partners had greater connection to the LGBT community than those who were bisexual/heterosexual. These factors all relate heavily to sexual identity. For example, heterosexual-identifying men who have sex with men many are often disconnected from sexual minority communities, in part due to internalized homophobia, and depression related to stigma [9, 36]. This disconnection from queer communities aligns with our findings. Given that these communities are often important venues for HIV prevention messaging, heterosexual men who have sex with men may be at greater HIV risk, which may explain their high representation in our sample, and highlights important HIV prevention and care needs.

Notably, LGBT community connectedness was associated with higher education and state differences, but not with other socioeconomic measures. It should be noted that our sample is specific to the 3 Mid-Atlantic jurisdictions that are uniquely socioeconomically diverse. Both employment type and housing here, particularly in Washington, D.C., are often transient [37]. These less stable socioeconomic measures may not fully capture longstanding socioeconomic contexts salient to intracommunity connection, though socioeconomic context is broadly related to HIV care nonetheless [1]. Our findings demonstrate not only the utility of the Connectedness to the LGBT Community Scale in Mid-Atlantic BSMMLWH, but also the unique nuances of this population’s social and economic context.

Our research has notable strengths. Our scale consisted of several items capturing many different aspects of LGBT community connectedness. Moreover, through factor analyses, we were able to confirm the unidimensionality of our scales in a consistent and data-driven manner. Additionally, our study delves into a pioneering approach to measuring a relatively overlooked concept within a significant demographic for health promotion endeavors: the connection to the LGBT community among Black SMMLWH. Enhancing our methodologies to capture this community context holds significance, particularly considering its ramifications for HIV prevention initiatives and the distinctive social environment of this group, such as HIV-related stigma and intersecting marginalized identities.

Our research also has important limitations that warrant comment. The focus on Mid-Atlantic Black SMMLWH does limit generalizability, as findings cannot be readily extrapolated to other populations. Given the importance of social connection to several health-related factors in this population, as evidenced by many of our identified criterion relationships, this focus is appropriate, however [14, 22, 23, 38]. Our measure was unidimensional based on our findings; while this is a strength for the overall measure, it preventing us from identifying possible subscales in a data-driven way. Finally, connection to the LGBT community and criterion measures were all self-reported, making social desirability bias a relevant factor. Despite this limitation, we were able to identify several forms of validity supporting the use of our measure of connection to the LGBT community in this population.

## Conclusion

Overall, the Connectedness to the LGBT Community Scale demonstrated strong validity in a sample of Black SMMLWH in the U.S. Mid-Atlantic region. Greater LGBT community connectedness was associated with greater social support and resilience, and lower internalized homophobia, hopelessness, depression, and HIV stigma. Future studies understanding the multi-level factors associated with HIV-related care outcomes in Black SMMLWH should consider the inclusion of the Connectedness to the LGBT Community Scale as a significant strengths-based construct promoting health and well-being in this highly marginalized population. Similarly, further explorations of relationships between community connection and intracommunity stigma are recommended, particularly in other cohort studies of Black SMM.

## Figures and Tables

**Table 1:** Sociodemographics and mean connectedness to the LGBT Community Scale among Mid-atlantic Black sexual minority men living with HIV (*n* = 650)

	Frequency	Percent	Connectedness to the LGBT Community Mean Score (SD)

**Age** ^1^			
18–24	30	4.6	**24.5 (6.3)**
25–34	304	46.8	**24.5 (6.9)**
35–44	298	45.9	**30.0 (7.3)**
45 and older	18	2.77	**27.3 (7.8)**
**State** ^2^			
Maryland	106	16.3	**28.0 (6.8)**
Pennsylvania	240	36.9	**32.4 (5.6)**
Washington, D.C.	304	46.8	**22.6 (6.2)**
**Education** ^1^			
Less than 4-year college	93	14.3	**23.6 (6.5)**
Undergraduate degree	319	49.1	**27.2 (7.2)**
Some graduate degree	94	14.5	**26.3 (8.3)**
Graduate degree	144	22.2	**29.5 (7.5)**
**Income** ^1^			
$20,000 to $40,000	56	8.6	28.4 (7.0)
$40,000 to $60,000	225	34.6	29.9 (7.5)
$60,000 to $80,000	209	32.2	22.6 (6.8)
$80,000 to $100,000	93	14.3	27.8 (6.1)
Over $100,000	67	10.3	29.5 (5.9)
**Stable Housing** ^3^			
No stable housing	93	14.3	26.8 (6.9)
Stable housing	557	85.7	27.1 (7.7)
**Sexual Partners** ^3^			
Men only	417	64.2	**30.8 (6.1)**
Men and women	233	35.9	**20.4 (4.7)**
**Sexual Identity** ^2^			
Bisexual / Queer	25	3.9	**23.4 (6.6)**
Gay / Same-gender loving	423	65.1	**30.8 (6.2)**
Straight / Heterosexual	202	31.1	**19.7 (3.4)**
**Sexual Role Preference** ^2^			
Bottom	392	60.3	**25.7 (7.9)**
Versatile	179	27.5	**28.7 (6.9)**
Top	79	12.2	**30.4 (5.2)**
**Relationship Status** ^3^			
Partnered	280	43.1	**29.9 (6.6)**
Single	370	56.9	**24.9 (7.5)**

1 Associations with Connectedness to the LGBT Community tested using Spearman’s rank-sum correlation. Bolded values indicate *p* < 0.05

2 Associations with Connectedness to the LGBT Community tested using Kruskal-Wallis test. Bolded values indicate *p* < 0.05

3 Associations with Connectedness to the LGBT Community tested using Cochran-Armitage test of trend. Bolded values indicate *p* < 0.05

**Table 2:** Connectedness to the LGBT Community Scale items and factor analysis among Mid-atlantic Black sexual minority men living with HIV (*n* = 650)

	Mean	Median	Standard Deviation	Factor 1

1. You feel you’re a part of the LGBT community.	3.07	3	0.97	0.84
2. Participating in the LGBT community is a positive thing for you.	3.02	3	0.99	0.86
3. You feel a bond with the LGBT community	3.06	3	0.98	0.84
4. You are proud of the LGBT community	3.05	3	1.01	0.86
5. It is important for you to be politically active in the LGBT community.	2.89	3	1.04	0.80
6. If we work together, gay, bisexual and lesbian people can solve problems in the LGBT community	3.04	3	0.99	0.85
7. You really feel that any problems faced by the LGBT community are also your own problems	2.97	3	1.03	0.85
8. You feel a bond with other gay men	3.02	3	0.97	0.81
9. You feel a bond with other others in the LGBT community.	2.97	3	0.99	0.86

Cronbach’s alpha = 0.948. Exploratory factor analysis yielded a single factor. Confirmatory factor analysis yielded a goodness of fit index = 1, a root mean square error of approximation < 0.01, and the single primary factor explaining > 80% of all variance across models with 1 to 4 fixed factors

All items were strongly positively correlated (All Spearman’s rank sum correlations > 0.70, all *p* < 0.001)

**Table 3:** Spearman’s rank-sum correlations between connectedness to the LGBT Community Scale and criterion measures among Mid-atlantic Black sexual minority men living with HIV (*n* = 650)

	Internalized Homophobia	Hopelessness	Depression	HIV Stigma	Social Support	Resilience

Connectedness to the LGBT Community	−0.77	−0.12	−0.58	−0.64	0.61	0.59

All correlations statistically significant (*p* < 0.05)

**Table 4:** Cumulative odds ratios for connectedness to the LGBT Community Scale quartiles and criterion measures among Mid-atlantic Black sexual minority men living with HIV (*n* = 650)

	Unadjusted			Adjusted*		
		
	COR	Lower CI	Upper CI	aCOR	Lower CI	Upper CI

Internalized Homophobia Scale	0.12	0.10	0.15	0.19	0.15	0.25
Hopelessness Scale	0.79	0.66	0.95	0.53	0.41	0.68
Depression Scale	0.30	0.26	0.35	0.61	0.50	0.75
HIV Stigma Scale	0.26	0.22	0.30	0.58	0.47	0.72
Social Support Scale	3.85	3.23	4.58	2.38	1.91	2.97
Resilience Scale	3.41	2.90	4.00	2.53	2.03	3.15

* Adjusted for age, state, education, income, housing status, gender of sexual partners, sexual position, and relationship status

All estimates statistically significant (*p* < 0.05)
